# An improved extension of Xgamma distribution: Its properties, estimation and application on failure time data

**DOI:** 10.1016/j.heliyon.2025.e41976

**Published:** 2025-01-15

**Authors:** Abdullah M. Alomair, Ayesha Babar, Muhammad Ahsan-ul-Haq, Saadia Tariq

**Affiliations:** a Department of Quantitative Methods, School of Business, King Faisal University, 31982, Al-Ahsa, Saudi Arabia; b School of Statistics, Minhaj University Lahore, Lahore, Pakistan; c College of Statistical Sciences, University of the Punjab, Lahore, Pakistan; d Quality Enhancement Cell, National College of Arts, Lahore, Pakistan

**Keywords:** Xgamma distribution, Power transformation, Entropy, Inference, Engineering data, Analysis

## Abstract

A new, more flexible model, the power quasi-Xgamma (PQXg) distribution, is introduced by adding an extra shape parameter using the power transformation approach. The PQXg distribution is more flexible due to its variable failure rate shapes. We derived its various theoretical properties including moments and its associated measures. Some reliability measurements include the survival function, hazard rate, mean residual life, Rényi and Tsallis entropy, and stress-strength reliability. Five approaches are used for parameter estimation: maximum likelihood, Anderson-Darling, Cramér-von Mises, ordinary, and weighted least squares. A Monte Carlo simulation is used to determine the effectiveness of these estimators for different sample sizes. The use of derived distribution is investigated utilizing symmetrical and asymmetric data sets from real-world fields. When compared to other competing distributions considered, the new distribution performed better.

## 1 Introduction

In the last few decades, it has become the most interesting task for researchers to model and analyze real-life data sets having complex behavior with maximum possible precision. To deal with real-life data sets related to various disciplines e.g., engineering, medicine, and social sciences having diverse behavior became the need of the day to derive new distributions that fit the data with more accuracy. Different techniques are utilized to make the base model more flexible which results in a new distribution. To enhance the flexibility of the existing distribution, one of the techniques is to add a new shape parameter. These techniques include the Marshall-Olkin (MO) family [1], length-biased truncated Lomax-G family [2], Type I Half Logistic Burr X-G family [3], new Topp Leone-G family [4], exponentiated-G family [5], Power Lindley-G family [6], Odd Fréchet-G family [7], truncated power Lomax-G family [8], Type II half logistic family [9], power transformation [10], transmuted family [11], and the mixture of two or more probability distributions [12]. Various probability models are proposed using these generalization approaches. Some examples are; MO exponential Weibull [13], MO inverted Kumaraswamy [14], MO length biased exponential [15], exponentiated Weibull [16], transmuted power function [17], exponentiated transmuted power function [18] and for brief survey reader can consult following references [19,20].

Variable power transformation has also gained attention in the last few years. Using this technique multiple models are introduced such as power half logistic distribution [21], power Lomax distribution [22], power Rayleigh distribution [23], power erlang distribution [24], power inverted Topp–Leone [25], and power inverted Nadarajah–Haghighi distribution [26].

In the present study, a new three-parameter quasi Xgamma distribution using power transformation. The following are the research goals of this study.

• To introduce a new probability distribution with an additional shape parameter. The new model is more flexible than the baseline distribution and can analyze datasets of different shapes decreasing, increasing, and unimodal.
• The second goal is to estimate the model parameters using five different estimation methods. Conduct a comprehensive simulation study to assess the behavior of derived estimators as well as to identify which estimation approach to estimate the model parameters efficiently.
• Two datasets from different fields are used to show the flexibility and applicability of the proposed distribution.

The rest of the paper is as; in section 2 the new distribution is proposed, and the flexibility of the new model is elaborated from a graphical presentation, section 3 deals with the mathematical properties of the distribution along with some reliability properties. Section 4 is devoted to the estimation of the parameters using the five different estimation methods. A simulation study is also executed in section 4 to elaborate on the performance of the parameter estimation methods. Section 5 deals with real-life applications to illustrate the performance of the distribution derived. Section 6 concludes the study.

## 2 Derivation of PQXg distribution

A random variable Z follows Quasi Xgamma (QXg) distribution [27]. The probability density function (PDF) of the QXg distribution is given below.

(1) $$f\left(z\right)=\frac{\lambda }{1+\eta }\left(\eta +\frac{\lambda^{2}}{2}z^{2}\right)\;e^{-\lambda z};\;z>0\text{,}$$

Now the new model is introduced using the power transformation to random variable Y $=Z^{1/\vartheta }$. The random variable Z belongs to the QXg distribution. The random variable Y then follows the QXg distribution denoted by Y∼(λ,η,ϑ) and abbreviated as the PQXg distribution. The cumulative distribution function (CDF) is given by

(2) $$F\left(y\right)=1-\frac{\left(1+\eta +\lambda y^{\vartheta }+\frac{\lambda^{2}y^{2\vartheta }}{2}\right)}{1+\eta }\;e^{-\lambda y^{\vartheta }},y>0\text{,}$$

and the density function of the PQXg distribution is

(3) $$f\left(y\right)=\frac{\lambda \vartheta y^{\vartheta -1}}{1+\eta }\left(\eta +\frac{\lambda^{2}y^{2\vartheta }}{2}\right)e^{-\lambda y^{\vartheta }},y>0\text{,}$$

where λ>0 is the scale parameter, η>0 and ϑ>0 are the shape parameters.

The asymptotic behavior of the PDF is described below.

(4) $$\lim_{y\to 0}f\left(y\right)=\left\{ \begin{array}{c} \infty for\;\vartheta <1\\ \frac{\eta \lambda }{1+\eta }\;for\;\vartheta =1\;\\ 0\;for\;\vartheta >1 \end{array}\right.$$

and

(5) $$\lim_{y\to \infty }f\left(y\right)=0\text{.}$$

**Fig. 1:**
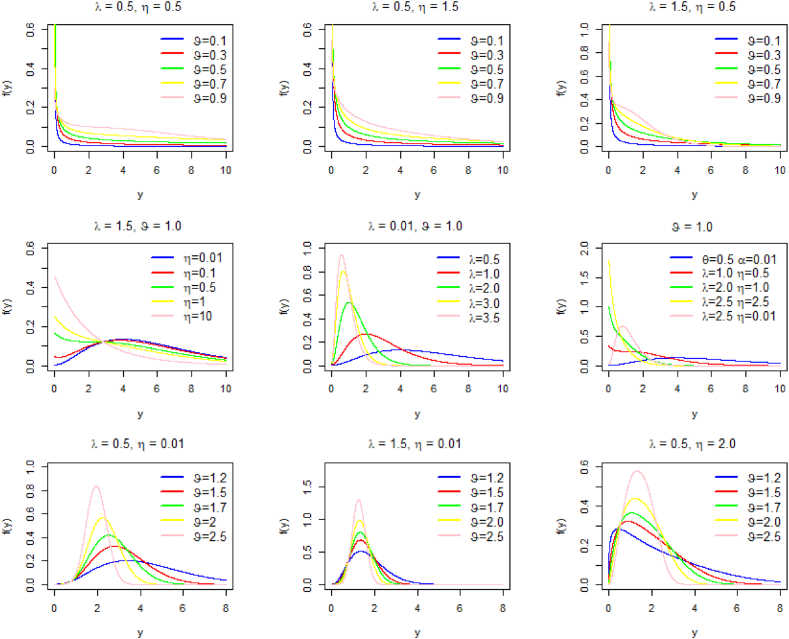
The PDF curves for some choices of parameters.

Fig. 1 illustrates graphically the different possible shapes of PDF of PQXg distribution, considering several combinations of parameters.

## 3 Theoretical properties

This section explores the mathematical properties of PQXg distribution.

### 3.1 Moments

The rth moments of the PQXg distribution are presented below.

(6) $$\mu_{r}^{\prime }=\frac{\Gamma \left(\frac{r}{\vartheta }+1\right)\;}{{\left(1+\eta \right)\lambda }^{\frac{r}{\vartheta }}}\left[\eta +\frac{1}{2}\;\left(\frac{r}{\vartheta }+2\right)\left(\frac{r}{\vartheta }+1\right)\right]\text{.}$$

Proof: Utilizing the PDF of the PQXg distribution

$$\mu_{r}^{\prime }=\frac{\lambda \vartheta }{1+\eta }\int_{0}^{\infty }y^{r+\vartheta -1}\;\left(\eta +\frac{\lambda^{2}y^{2\vartheta }}{2}\right)e^{-\lambda y^{\vartheta }}dy\text{,}$$

Substituting $\lambda y^{\vartheta }=x$, we obtain

$$\mu_{r}^{\prime }=\frac{1}{{\left(1+\eta \right)\lambda }^{\frac{r}{\vartheta }}}\int_{0}^{\infty }x^{\frac{r}{\vartheta }}\;\left(\eta +\frac{x^{2}}{2}\right)e^{-x}dx\text{,}$$

$$=\frac{1}{{\left(1+\eta \right)\lambda }^{\frac{r}{\vartheta }}}\left[\eta \int_{0}^{\infty }x^{\frac{r}{\vartheta }}\;e^{-x}dz+\frac{1}{2}\int_{0}^{\infty }x^{\frac{r}{\vartheta }+2}\;e^{-x}dx\right]\text{,}$$

$$=\frac{\Gamma \left(\frac{r}{\vartheta }+1\right)\;}{{\left(1+\eta \right)\lambda }^{\frac{r}{\vartheta }}}\left[\eta +\frac{1}{2}\left(\frac{r}{\vartheta }+2\right)\left(\frac{r}{\vartheta }+1\right)\right]\text{.}$$

To obtain the ordinary moments of PQXg distribution substitute r=1,2,3, and 4 as below

$$\mu_{1}^{\prime }=\frac{\Gamma \left(\frac{1}{\vartheta }+1\right)\;}{{\left(1+\eta \right)\lambda }^{\frac{1}{\vartheta }}}\left[\eta +\frac{1}{2}\left(\frac{1}{\vartheta }+2\right)\left(\frac{1}{\vartheta }+1\right)\right]\text{,}$$

$$\mu_{2}^{'}=\frac{\Gamma \left(\frac{2}{\vartheta }+1\right)\;}{{\left(1+\eta \right)\lambda }^{\frac{2}{\vartheta }}}\left[\eta +\frac{1}{2}\left(\frac{2}{\vartheta }+2\right)\left(\frac{2}{\vartheta }+1\right)\right]\text{,}$$

$$\mu_{3}^{\prime }=\frac{\Gamma \left(\frac{3}{\vartheta }+1\right)\;}{{\left(1+\eta \right)\lambda }^{\frac{3}{\vartheta }}}\left[\eta +\frac{1}{2}\left(\frac{3}{\vartheta }+2\right)\left(\frac{3}{\vartheta }+1\right)\right]\text{,}$$

and

$$\mu_{4}^{\prime }=\frac{\Gamma \left(\frac{4}{\vartheta }+1\right)\;}{{\left(1+\eta \right)\lambda }^{\frac{4}{\vartheta }}}\left[\eta +\frac{1}{2}\left(\frac{4}{\vartheta }+2\right)\left(\frac{4}{\vartheta }+1\right)\right]\text{.}$$

The variance of PQXg distribution is

$$Var\left(Y\right)=\mu_{2}^{\prime }-{\left(\mu_{1}^{\prime }\right)}^{2}$$

$$=\frac{\Gamma \left(\frac{2}{\vartheta }+1\right)\;}{{\left(1+\eta \right)\lambda }^{\frac{2}{\vartheta }}}\left[\eta +\frac{1}{2}\left(\frac{2}{\vartheta }+2\right)\left(\frac{2}{\vartheta }+1\right)\right]-{\left(\frac{\Gamma \left(\frac{1}{\vartheta }+1\right)\;}{{\left(1+\eta \right)\lambda }^{\frac{1}{\vartheta }}}\left[\eta +\frac{1}{2}\left(\frac{1}{\vartheta }+2\right)\left(\frac{1}{\vartheta }+1\right)\right]\right)}^{2}\text{.}$$

The coefficient of skewness (CS), coefficient of kurtosis (CK), and dispersion index (DI) may be calculated using.

$$\text{CS}=\frac{\mu_{3}^{\prime }-3\mu_{2}^{\prime }\mu_{1}^{\prime }+2{\left({\mu^{\prime }}_{1}\right)}^{3}}{{\left({\mu^{\prime }}_{2}-{\left({\mu^{\prime }}_{1}\right)}^{2}\right)}^{\frac{3}{2}}},\text{CK}=\frac{\mu_{4}^{\prime }{-4\mu }_{3}^{\prime }\mu_{1}^{\prime }+6\mu_{2}^{\prime }{\left({\mu^{\prime }}_{2}\right)}^{2}-3{\left({\mu^{\prime }}_{1}\right)}^{4}}{{\left({\mu^{\prime }}_{2}-{\left({\mu^{\prime }}_{1}\right)}^{2}\right)}^{2}}\;\text{and}\;\text{DI}=\frac{Var\left(Y\right)}{Mean}\text{.}$$

From Table 1, the numerical results obtained showed that the density curves are positively skewed and leptokurtic for ϑ<1. The model is behaving approximately normal for ϑ>1. The probability model is versatile and applicable to various datasets exhibiting the specified behavior.

**Table 1:** Some descriptive measures of PQXg distribution for (λ = 1.5, η = 0.5).

ϑ	E(Y)	Var	CS	CK	DI
0.1	4.6800	220.25	4.0234	19.8043	47.0629
0.3	9.7759	313.50	2.6285	10.1121	32.0683
0.5	3.8102	34.111	3.5753	24.2010	8.9523
0.7	2.1667	5.6894	2.0322	9.7267	2.6259
0.9	1.6569	2.1541	1.2685	5.4734	1.3000
1.0	1.5234	1.5304	1.0124	4.5311	1.0046
1.2	1.3584	0.9042	0.6300	3.5516	0.6656
1.4	1.2624	0.6078	0.3612	3.1610	0.4815
1.6	1.1980	0.4429	0.1852	3.0434	0.3697
1.8	1.1456	0.3414	0.1201	3.1418	0.2980
2.0	1.0943	0.2682	0.1592	3.6621	0.2451

### 3.2 Moment-generating function

The moment-generating function is obtained and presented below.

$$M_{0}\left(t\right)=\int_{0}^{\infty }e^{ty}f\left(y\right)dy=\int_{0}^{\infty }e^{ty}\;\frac{\lambda \vartheta y^{\vartheta -1}}{1+\eta }\left(\eta +\frac{\lambda^{2}y^{2\vartheta }}{2}\right)e^{-\lambda y^{\vartheta }}\text{,}$$

$$=\frac{\lambda \vartheta }{1+\eta }\int_{0}^{\infty }y^{\vartheta -1}\;\left(\eta +\frac{\lambda^{2}y^{2\vartheta }}{2}\right)e^{-\lambda y^{\vartheta }+ty}dy\text{,}$$

using $e^{y}=\sum_{r=0}^{\infty }\frac{y^{r}}{r!}$

$$M_{0}\left(t\right)=\frac{\lambda \vartheta }{1+\eta }\sum_{r=0}^{\infty }\frac{t^{r}}{r!}\;\int_{0}^{\infty }y^{r+\vartheta -1}\;\left(\eta +\frac{\lambda^{2}y^{2\vartheta }}{2}\right)e^{-\lambda y^{\vartheta }}dy\text{,}$$

(7) $$=\frac{1}{\left(1+\eta \right)\lambda^{\frac{r}{\vartheta }}}\sum_{r=0}^{\infty }\frac{t^{r}}{r!}\Gamma \left(\frac{r}{\vartheta }+1\right)\left[\eta +\frac{1}{2}\left(\frac{r}{\vartheta }+2\right)\left(\frac{r}{\vartheta }+1\right)\right]\text{.}$$

### 3.3 Incomplete moments

The incomplete moments are important and utilized to derive many properties. The incomplete moments for PQXg distribution can be derived as

$$\phi_{r}=\int_{0}^{t}y^{r}\frac{\lambda \vartheta y^{\vartheta -1}}{1+\eta }\left(\eta +\frac{\lambda^{2}y^{2\vartheta }}{2}\right)e^{-\lambda y^{\vartheta }}dy\text{,}$$

$$=\frac{1}{1+\eta }\int_{0}^{t}y^{r}\;\left(\eta +\frac{\lambda^{2}y^{2\vartheta }}{2}\right)e^{-\lambda y^{\vartheta }}\left(\lambda \vartheta y^{\vartheta -1}\right)dy\text{,}$$

Let $\lambda y^{\vartheta }=z$

$$\phi_{r}=\frac{1}{{\left(1+\eta \right)\lambda }^{\frac{r}{\vartheta }}}\int_{0}^{\lambda t^{\vartheta }}z^{\frac{r}{\vartheta }}\;\left(\eta +\frac{z^{2}}{2}\right)e^{-z}dz\text{,}$$

$$=\frac{1}{{\left(1+\eta \right)\lambda }^{\frac{r}{\vartheta }}}\left[\eta \int_{0}^{\lambda t^{\vartheta }}z^{\frac{r}{\vartheta }}\;e^{-z}dz+\frac{1}{2}\int_{0}^{\lambda t^{\vartheta }}z^{\frac{r}{\vartheta }+2}\;e^{-z}dz\right]\text{,}$$

(8) $$=\frac{1}{{\left(1+\eta \right)\lambda }^{\frac{r}{\vartheta }}}\left[\eta \Gamma \left(\frac{r}{\vartheta }+1,\lambda t^{\vartheta }\right)+\frac{\Gamma \left(\frac{r}{\vartheta }+3,\lambda t^{\vartheta }\right)}{2}\right]\text{.}$$

### 3.4 Quantile function

To find the ordered points of the data set, the function is used to be known as the quantile function. The quantile function can be obtained using relation F(y)=R and solve it for y.

(9) $$\left(1+\eta +\lambda y^{\vartheta }+\frac{\lambda^{2}y^{2\vartheta }}{2}\right)\;e^{-\lambda y^{\vartheta }}=\left(1-R\right)\left(1+\eta \right)\text{.}$$

The above equation cannot be expressed explicitly in terms of y, so R software will be used to generate random numbers.

### 3.5 Rényi entropy

The Rényi entropy is defined for a probability distribution as:

$$H_{R}\left(\delta \right)=\frac{1}{1-\delta }\ln\left[\int_{0}^{\infty }{\left(f\left(y\right)\right)}^{\delta }dy\right],\delta >0,\delta \neq 1\text{.}$$

The Rényi entropy for PQXg distribution is derived as

$$\int_{0}^{\infty }{\left(f\left(y\right)\right)}^{\delta }dy=\frac{{\left(\lambda \vartheta \eta \right)}^{\delta }}{{\left(1+\eta \right)}^{\delta }}\int_{0}^{\infty }{\left(1+\frac{\lambda^{2}}{2\eta }y^{2\vartheta }\right)}^{\delta }y^{\delta \left(\vartheta -1\right)}e^{-\lambda \delta y^{\vartheta }}dy\text{,}$$

using the following binomial expansion ${\left(1+z\right)}^{n}=\sum_{i=0}^{\infty }\left(\begin{array}{c} n\\ i \end{array}\right)z^{i}\text{,}$ the expression becomes

$$\int_{0}^{\infty }{\left(f\left(y\right)\right)}^{\delta }dy=\frac{{\left(\lambda \vartheta \eta \right)}^{\delta }}{{\left(1+\eta \right)}^{\delta }}\sum_{i=0}^{\infty }\left(\begin{array}{c} \delta \\ i \end{array}\right)\frac{\lambda^{2i}}{{\left(2\eta \right)}^{i}}\int_{0}^{\infty }y^{2\vartheta i+\delta \left(\vartheta -1\right)}e^{-\lambda \delta y^{\vartheta }}dy\text{,}$$

substitute $\lambda \delta y^{\vartheta }=t$,

$$\int_{0}^{\infty }{\left(f\left(y\right)\right)}^{\delta }dy=\frac{{\left(\lambda \vartheta \eta \right)}^{\delta }}{\vartheta {\left(1+\eta \right)}^{\delta }}\sum_{i=0}^{\infty }\left(\begin{array}{c} \delta \\ i \end{array}\right)\frac{\lambda^{2i}}{{\left(2\eta \right)}^{i}}\frac{1}{{\left(\lambda \delta \right)}^{\frac{2\vartheta i+\left(\delta -1\right)\left(\vartheta -1\right)}{\vartheta }+1}}\int_{0}^{\infty }t^{\frac{2i\vartheta +\left(\delta -1\right)\left(\vartheta -1\right)}{\vartheta }}e^{-t}dt\text{,}$$

$$=\frac{{\left(\lambda \vartheta \eta \right)}^{\delta }}{\vartheta {\left(1+\eta \right)}^{\delta }}\sum_{i=0}^{\infty }\frac{\left(\begin{array}{c} \delta \\ i \end{array}\right)\lambda^{2i}}{{\left(2\eta \right)}^{i}{\left(\lambda \delta \right)}^{\frac{2\vartheta i+\left(\delta -1\right)\left(\vartheta -1\right)}{\vartheta }+1}}\Gamma \left(\frac{2i\vartheta +\left(\delta -1\right)\left(\vartheta -1\right)}{\vartheta }+1\right)\text{,}$$

hence

(10) $$H_{R}\left(\delta \right)=\frac{1}{1-\delta }\ln\left[\frac{{\left(\lambda \vartheta \eta \right)}^{\delta }}{\vartheta {\left(1+\eta \right)}^{\delta }}\sum_{i=0}^{\infty }\frac{\left(\begin{array}{c} \delta \\ i \end{array}\right)\lambda^{2i}}{{\left(2\eta \right)}^{i}{\left(\lambda \delta \right)}^{\frac{2\vartheta i+\left(\delta -1\right)\left(\vartheta -1\right)}{\vartheta }+1}}\Gamma \left(\frac{2i\vartheta +\left(\delta -1\right)\left(\vartheta -1\right)}{\vartheta }+1\right)\;\right]\;\text{.}$$

### 3.6 Tsallis entropy

The Tsallis entropy is defined for probability distribution as.

$$S_{p}\left(y\right)=\frac{1}{p-1}\ln\left[1-\int_{0}^{\infty }{\left(f\left(y\right)\right)}^{p}dy\right],p>0,p\neq 1$$

Consider the integral part

$$\int_{0}^{\infty }{\left(f\left(y\right)\right)}^{p}dy=\frac{{\left(\lambda \vartheta \eta \right)}^{p}}{{\vartheta \left(1+\eta \right)}^{p}}\sum_{i=1}^{\infty }\frac{\left(\begin{array}{c} p\\ i \end{array}\right)\lambda^{2i}}{{\left(2\eta \right)}^{i}{\left(\lambda p\right)}^{\frac{2i\vartheta +\left(p-1\right)\left(\vartheta -1\right)}{\vartheta }+1}}\Gamma \left(\frac{2i\vartheta +\left(p-1\right)\left(\vartheta -1\right)}{\vartheta }+1\right)\text{,}$$

and

(11) $$S_{p}\left(y\right)=\frac{1}{p-1}\ln\left[\frac{{\left(\lambda \vartheta \eta \right)}^{p}}{{\vartheta \left(1+\eta \right)}^{p}}\sum_{i=1}^{\infty }\frac{\left(\begin{array}{c} p\\ i \end{array}\right)\lambda^{2i}}{{\left(2\eta \right)}^{i}{\left(\lambda p\right)}^{\frac{2i\vartheta +\left(p-1\right)\left(\vartheta -1\right)}{\vartheta }+1}}\Gamma \left(\frac{2i\vartheta +\left(p-1\right)\left(\vartheta -1\right)}{\vartheta }+1\right)\right]\text{.}$$

### 3.7 Reliability analysis

This section focuses on the derivation of some key reliability characteristics.

The survival function is

$$S\left(y\right)=\frac{\left(1+\eta +\lambda y^{\vartheta }+\frac{\lambda^{2}y^{2\vartheta }}{2}\right)}{1+\eta }\;e^{-\lambda y^{\vartheta }}\text{.}$$

The hazard rate of PQXg distribution is

(12) $$h\left(y\right)=\frac{\lambda {\vartheta y}^{\vartheta -1}\left(\eta +\frac{\lambda^{2}y^{2\vartheta }}{2}\right)}{\left(1+\eta +\lambda y^{\vartheta }+\frac{\lambda^{2}y^{2\vartheta }}{2}\right)\;}\text{.}$$

The asymptotic behavior of the hazard function is described below.

$\lim_{y\to 0}h\left(y\right)=\left\{ \begin{array}{c} \infty \vartheta <1\\ \frac{\lambda \eta }{1+\eta \;}\;\vartheta =1\\ \;0\;\vartheta >1\; \end{array}\right.$ and $\lim_{y\to \infty }h\left(y\right)=\left\{ \begin{array}{c} 0\;\vartheta <1\\ \lambda \;\vartheta =1\\ \infty \vartheta >1\; \end{array}\right.$

**Fig. 2:**
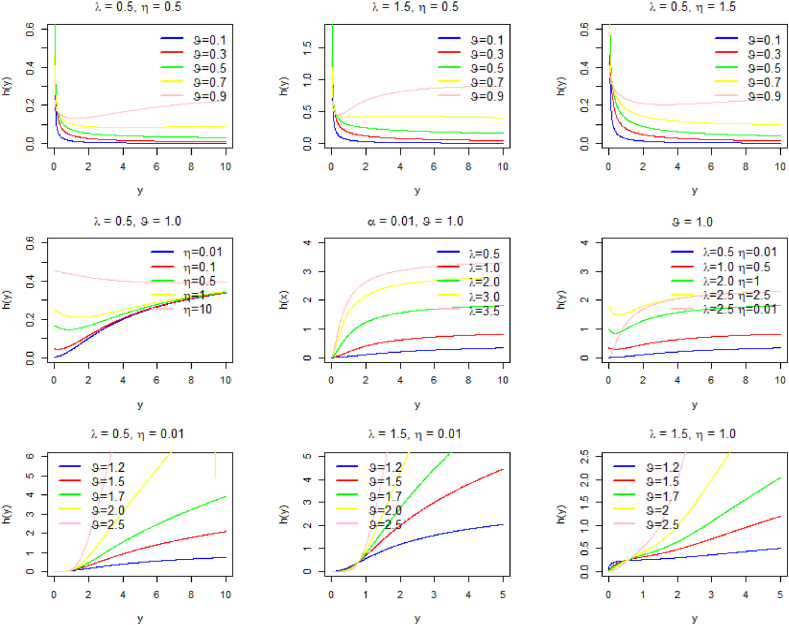
The hazard curves for some choices of parameters.

The expression above shows that the hazard function of the PQXg distribution falls when ϑ<1, is constant when ϑ=1, and grows when ϑ>1. This pattern is clearly shown in Fig. 2.

The reversed hazard function is

$$r\left(y\right)=\frac{\lambda \vartheta y^{\vartheta -1}\left(\eta +\frac{\lambda^{2}y^{2\vartheta }}{2}\right)e^{-\lambda y^{\vartheta }}}{\left(1+\eta \right)-\left(1+\eta +\lambda y^{\vartheta }+\frac{\lambda^{2}y^{2\vartheta }}{2}\right)e^{-\lambda y^{\vartheta }}}\text{.}$$

The second failure rate of PQXg distribution is

$$r^{*}\left(y\right)=\log\left[\frac{\left(1+\eta +\theta y^{\vartheta }+\frac{\lambda^{2}y^{2\vartheta }}{2}\right)e^{-\lambda y^{\vartheta }}}{\left(1+\eta +\lambda {\left(y+1\right)}^{\vartheta }+\frac{\lambda^{2}}{2}{\left(y+1\right)}^{2\vartheta }\right)e^{-\lambda {\left(y+1\right)}^{\vartheta }}}\right]\text{.}$$

### 3.8 Mean residual life

The Mean Residual Life (MRL) is used to estimate the average remaining lifetime of an object. If Y represents the lifetime of the object, the MRL is expressed as:

$$MRL=E\left(Y-t|Y>t\right)=\frac{1}{1-F\left(t\right)}\;\int_{t}^{\infty }\left(1-F\left(y\right)\right)dy\text{,}$$

$$=\frac{1}{\left(1+\eta +\lambda t^{\vartheta }+\frac{\lambda^{2}t^{2\vartheta }}{2}\right)e^{-\lambda t^{\vartheta }}}\;\left[\int_{t}^{\infty }\left(1+\eta +\lambda y^{\vartheta }+\frac{\lambda^{2}y^{2\vartheta }}{2}\right)\;e^{-\lambda y^{\vartheta }}dy\right]\text{,}$$

$$=\frac{1}{\left(1+\eta +\lambda t^{\vartheta }+\frac{\lambda^{2}t^{2\vartheta }}{2}\right)e^{-\lambda t^{\vartheta }}}\;\left[\left(1+\eta \right)\int_{t}^{\infty }\;e^{-\lambda y^{\vartheta }}dy+\lambda \int_{t}^{\infty }y^{\vartheta }\;e^{-\lambda y^{\vartheta }}dy+\frac{\lambda^{2}}{2}\int_{t}^{\infty }y^{2\vartheta }\;e^{-\lambda y^{\vartheta }}dy\right]\text{,}$$

substitute $\lambda y^{\vartheta }=z\Rightarrow y^{\vartheta }=\frac{z}{\lambda },dy=\frac{dz}{\lambda \vartheta y^{\vartheta -1}}\Rightarrow dt=\frac{z^{\frac{1}{\vartheta }}dz}{\vartheta z}$

$$MRL=\frac{1}{\left(1+\eta +\lambda t^{\vartheta }+\frac{\lambda^{2}t^{2\vartheta }}{2}\right)e^{-\lambda t^{\vartheta }}}\;\left[\left(1+\eta \right)\int_{\lambda t^{\vartheta }}^{\infty }\;e^{-z}\frac{z^{\frac{1}{\vartheta }}dz}{\vartheta z}+\lambda \int_{\lambda t^{\vartheta }}^{\infty }\frac{z}{\lambda }\;e^{-z}\frac{z^{\frac{1}{\vartheta }}dz}{\vartheta z}+\frac{\lambda^{2}}{2}\int_{\lambda t^{\vartheta }}^{\infty }\frac{z^{2}}{\lambda^{2}}\;e^{-z}\frac{z^{\frac{1}{\vartheta }}dz}{\vartheta z}\right]\text{,}$$

$$=\frac{\left[\frac{\left(1+\eta \right)}{\vartheta }\Gamma \left(\frac{1}{\vartheta },\lambda t^{\vartheta }\right)+\frac{1}{\vartheta }\Gamma \left(\frac{1}{\vartheta }+1,\lambda t^{\vartheta }\right)+\frac{1}{2\vartheta }\Gamma \left(\frac{1}{\vartheta }+2,\lambda t^{\vartheta }\right)\right]}{\left(1+\eta +\lambda t^{\vartheta }+\frac{\lambda^{2}t^{2\vartheta }}{2}\right)e^{-\lambda t^{\vartheta }}}\text{.}$$

### 3.9 Stress strength reliability (SSR)

The SSR, when stress follows the $PQXg\;\left(y_{1};\lambda_{1},\eta_{1},\vartheta \right)$ and strength also follows $PQXg\;\left(y_{2};\lambda_{2},\eta_{2},\vartheta \right)$. The SSR for PQXg distribution is derived as follows.

$$R=\int_{0}^{\infty }\frac{\lambda_{1}{\vartheta y}^{\vartheta -1}}{1+\eta_{1}}\left(\eta_{1}+\frac{{\lambda_{1}}^{2}y^{2\vartheta }}{2}\right)e^{-\lambda_{1}y^{\vartheta }}\left[1-\frac{\left(1+\eta_{2}+\lambda_{2}x^{\vartheta }+\frac{\lambda_{2}^{2}y^{2\vartheta }}{2}\right)}{1+\eta_{2}}\;e^{-\lambda_{2}y^{\vartheta }}\right]dy\text{,}$$

$$=1-\frac{\lambda_{1}\vartheta }{\left(1+\eta_{1}\right)\left(1+\eta_{2}\right)}\int_{0}^{\infty }y^{\vartheta -1}\left(\eta_{1}+\frac{{\lambda_{1}}^{2}y^{2\vartheta }}{2}\right)\left(1+\eta_{2}+\lambda_{2}y^{\vartheta }+\frac{\lambda_{2}^{2}y^{2\vartheta }}{2}\right)\;e^{-\left({\lambda_{1}+\lambda }_{2}\right)y^{\vartheta }}dy\text{,}$$

let $\left({\lambda_{1}+\lambda }_{2}\right)y^{\vartheta }=t\Rightarrow \;y^{\vartheta }=\frac{t}{\left({\lambda_{1}+\lambda }_{2}\right)},\vartheta y^{\vartheta -1}dy=\frac{dt}{\left({\lambda_{1}+\lambda }_{2}\right)}$

$$R=1-\frac{\lambda_{1}}{\left(1+\eta_{1}\right)\left(1+\eta_{2}\right)}\left\{\frac{\eta_{1}\left(1+\eta_{2}\right)}{\left({\lambda_{1}+\lambda }_{2}\right)}\int_{0}^{\infty }e^{-t}dt+\frac{\eta_{1}\lambda_{2}}{{\left({\lambda_{1}+\lambda }_{2}\right)}^{2}}\int_{0}^{\infty }te^{-t}dt+\frac{{\eta_{1}\lambda }_{2}^{2}}{2{\left({\lambda_{1}+\lambda }_{2}\right)}^{3}}\int_{0}^{\infty }t^{2}e^{-t}dt+\left(1+\eta_{2}\right)\frac{\lambda_{1}^{2}}{2{\left({\lambda_{1}+\lambda }_{2}\right)}^{3}}\int_{0}^{\infty }t^{2}e^{-t}dt+\frac{\lambda_{1}^{2}\lambda_{2}}{2{\left({\lambda_{1}+\lambda }_{2}\right)}^{4}}\int_{0}^{\infty }t^{3}e^{-t}dt+\frac{\lambda_{1}^{2}\lambda_{2}^{2}}{4{\left({\lambda_{1}+\lambda }_{2}\right)}^{5}}\int_{0}^{\infty }t^{4}e^{-t}dt\;\right\}\text{,}$$

$$=1-\frac{\lambda_{1}}{\left(1+\eta_{1}\right)\left(1+\eta_{2}\right)}\left\{\frac{\eta_{1}\left(1+\eta_{2}\right)}{\left({\lambda_{1}+\lambda }_{2}\right)}+\frac{\eta_{1}\lambda_{2}}{{\left({\lambda_{1}+\lambda }_{2}\right)}^{2}}+\frac{{\eta_{1}\lambda }_{2}^{2}}{{\left({\lambda_{1}+\lambda }_{2}\right)}^{3}}+\frac{\lambda_{1}^{2}\left(1+\eta_{2}\right)}{{\left({\lambda_{1}+\lambda }_{2}\right)}^{3}}+\frac{3\lambda_{1}^{2}\lambda_{2}}{{\left({\lambda_{1}+\lambda }_{2}\right)}^{4}}+\frac{6\lambda_{1}^{2}\lambda_{2}^{2}}{{\left({\lambda_{1}+\lambda }_{2}\right)}^{5}}\right\}\text{.}$$

### 3.10 Mean inactivity time

The Mean Inactivity Time (MIT) function is utilized to estimate the expected duration of idle time that has elapsed. It is defined as:

$$m\left(t\right)=t-\frac{1}{G\left(t\right)}\int_{0}^{t}yg\left(y\right)dy,t>0\text{,}$$

$$\int_{0}^{t}yg\left(y\right)dy=\frac{\lambda \vartheta }{1+\eta }\int_{0}^{t}y^{\vartheta }\left(\eta +\frac{\lambda^{2}y^{2\vartheta }}{2}\right)e^{-\lambda y^{\vartheta }}dy\text{,}$$

*let* $\lambda y^{\vartheta }=z$

$$\int_{0}^{t}yg\left(y\right)dy=\frac{1}{1+\eta }\int_{0}^{\lambda y^{\vartheta }}{\left(\frac{z}{\lambda }\right)}^{\frac{1}{\vartheta }}\left(\eta +\frac{z^{2}}{2}\right)e^{-z}dz\text{,}$$

$$=\frac{1}{1+\eta }\left[\frac{\eta }{\lambda^{\frac{1}{\vartheta }}}\int_{0}^{\lambda y^{\vartheta }}z^{\frac{1}{\vartheta }}e^{-z}dz+\frac{1}{2\lambda^{\frac{1}{\vartheta }}}\int_{0}^{\lambda y^{\vartheta }}z^{\frac{1}{\vartheta }+2}e^{-z}dz\right]\text{,}$$

$$=\frac{1}{\left(1+\eta \right)\lambda^{\frac{1}{\vartheta }}}\left[\eta \Gamma \left(\frac{1}{\vartheta }+1,\lambda t^{\vartheta }\right)+\frac{\Gamma \left(\frac{1}{\vartheta }+3,\lambda t^{\vartheta }\right)}{2}\right]\text{,}$$

hence

$$m\left(t\right)=t-\frac{\left[\eta \Gamma \left(\frac{1}{\vartheta }+1,\lambda t^{\vartheta }\right)+\frac{\Gamma \left(\frac{1}{\vartheta }+3,\lambda t^{\vartheta }\right)}{2}\right]}{\lambda^{\frac{1}{\vartheta }}\left[\left(1+\eta \right)-\left(1+\eta +\lambda t^{\vartheta }+\frac{\lambda^{2}t^{2\vartheta }}{2}\right)\;e^{-\lambda t^{\vartheta }}\right]}\text{.}$$

### 3.11 Strong mean inactivity time

The Strong Mean Inactivity Time (SMIT) is described as:

$$M\left(t\right)=t^{2}-\frac{1}{G\left(t\right)}\int_{0}^{t}y^{2}g\left(y\right)dy,t>0\text{,}$$

consider $\int_{0}^{t}y^{2}g\left(y\right)dy=\frac{\lambda \vartheta }{1+\eta }\int_{0}^{t}y^{\vartheta +1}\left(\eta +\frac{\lambda^{2}y^{2\vartheta }}{2}\right)e^{-\lambda y^{\vartheta }}dy\text{,}$

using $\lambda y^{\vartheta }=z$*,*

$$\int_{0}^{t}y^{2}g\left(y\right)dy=\frac{1}{\lambda^{\frac{2}{\vartheta }}\left(1+\eta \right)}\left[\eta \int_{0}^{\lambda t^{\vartheta }}z^{\frac{2}{\vartheta }}e^{-z}dz+\frac{1}{2}\int_{0}^{\lambda t^{\vartheta }}z^{\frac{2}{\vartheta }+2}e^{-z}dz\right]\text{,}$$

$$=\frac{1}{\left(1+\eta \right)\lambda^{\frac{2}{\vartheta }}}\left[\eta \Gamma \left(\frac{2}{\beta }+1,\lambda t^{\vartheta }\right)+\frac{1}{2}\Gamma \left(\frac{2}{\vartheta }+3,\lambda t^{\vartheta }\right)\right]\text{,}$$

hence

$$M\left(t\right)=t^{2}-\frac{\left[\eta \Gamma \left(\frac{2}{\vartheta }+1,\lambda t^{\vartheta }\right)+\frac{1}{2}\Gamma \left(\frac{2}{\vartheta }+3,\lambda t^{\vartheta }\right)\right]}{\lambda^{\frac{2}{\vartheta }}\left[\left(1+\eta \right)-\left(1+\eta +\lambda t^{\vartheta }+\frac{\lambda^{2}t^{2\vartheta }}{2}\right)\;e^{-\lambda t^{\vartheta }}\right]}\text{.}$$

## 4 Estimation of parameters

In this section, we estimate the parameters of the PQXg distribution using five distinct estimation methods. The effectiveness of these methods is evaluated through extensive simulation study.

### 4.1 Maximum likelihood estimation (MLE)

Let $y_{1},y_{2},y_{3},\ldots ,y_{n}$ be a random sample of size n from PQXg distribution, then the log-likelihood function of maximum likelihood is as follows

(14) $$\ln\left(L\right)=n\;\log\;\lambda +n\;\log\;\vartheta +\left(\vartheta -1\right)\sum_{i=1}^{n}\ln\left(y_{i}\right)+\sum_{i=1}^{n}\ln\left(\eta +\frac{\lambda^{2}y_{i}^{2\vartheta }}{2}\right)-\lambda \;\sum_{i=1}^{n}y_{i}^{\vartheta }-n\;\ln\left(1+\eta \right)\text{.}$$

To estimate the unknown parameter values, we differentiate Eq. (14) with respect to the parameters and set the resulting equations to zero, yielding:

(15) $$\frac{\partial \ln\left(L\right)}{\partial \eta }=\sum_{i=1}^{n}\frac{1}{\left(\eta +\frac{\lambda^{2}y_{i}^{2\vartheta }}{2}\right)}-\frac{n}{\left(1+\eta \right)}=0\text{,}$$

(16) $$\frac{\partial \ln\left(L\right)}{\partial \lambda }=\frac{n}{\lambda }+\sum_{i=1}^{n}\frac{2\lambda y_{i}^{2\vartheta }}{\left(\eta +\frac{\lambda^{2}y_{i}^{2\vartheta }}{2}\right)}-\sum_{i=1}^{n}y_{i}^{\vartheta }=0\text{,}$$

(17) $$\frac{\partial \ln\left(L\right)}{\partial \vartheta }=\frac{n}{\vartheta }+\sum_{i=1}^{n}\ln\left(y_{i}\right)+\sum_{i=1}^{n}\frac{\lambda^{2}y_{i}^{2\vartheta }\;\ln\left(y_{i}\right)}{\left(\eta +\frac{\lambda^{2}y_{i}^{2\vartheta }}{2}\right)}-\lambda \;\sum_{i=1}^{n}y_{i}^{\vartheta }\;\ln\left(y_{i}\right)=0\text{.}$$

equations (15), (16), (17) are quite complicated and cannot be expressed explicitly for the values of parameters. To have a unique solution of unknown parameters, R software is used.

### 4.2 Anderson–Darling estimation (ADE)

The Anderson–Darling is a minimum distance-based estimation approach. The ADE approach is based on sample order values and the estimates can be obtained by minimizing the following statistics.

(18) $$ADE=-n-\frac{1}{n}\sum_{i=1}^{n}\left(2i-1\right)\left[\log\;F\left(y_{i:n}\right)+\log\;S\left(y_{i;n}\right)\right]\text{.}$$

### 4.3 Cramer von Mises estimation (CVME)

The CVME approach is also a minimum distance-based estimation approach and was originally proposed by Ref. [28]. The estimates can be obtained by minimizing the following statistics.

(19) $$ADE=\frac{1}{12n}+\sum_{i=1}^{n}{\left[F\left(y_{i:n}\right)-\frac{2i-1}{2n}\right]}^{2}\text{.}$$

### 4.4 Ordinary least-squares estimation (OLSE)

The OLS estimators were also attained by minimizing the following statistics:

(20) $$OLSE=\sum_{i=1}^{n}{\left[F\left(y_{i:n}\right)-\frac{i}{n+1}\right]}^{2}\text{.}$$

### 4.5 Weighted least-squares estimation (WLSE)

The WLS estimators are achieved by minimizing the following statistics

(21) $$WLSE=\sum_{i=1}^{n}{\frac{\left(n+2\right){\left(n+1\right)}^{2}}{i\left(n-i+1\right)}\left[F\left(y_{i:n}\right)-\frac{i}{n+1}\right]}^{2}\text{.}$$

### 4.6 Simulation study

To analyze the performance of PQXg distribution, the Monte Carlo simulation study is conducted with N = 10000 repetitions, by considering distinct values of sample n = 20, 50, 100, 200, and 300. To conduct the study, we have considered different combinations of parameters from the PQXg distribution. Comparing the performance of all derived estimators we compute the absolute bias (AB) and mean squared error (MSE) criteria.

$$AB\left(\eta \right)=\frac{1}{10000}\sum_{i=1}^{10000}\left|\hat{\eta }-\eta \right|,AB\left(\lambda \right)=\frac{1}{10000}\sum_{i=1}^{10000}\left|\hat{\lambda }-\lambda \right|\;and\;AB\left(\vartheta \right)=\frac{1}{10000}\sum_{i=1}^{10000}\left|\hat{\vartheta }-\vartheta \right|$$

and

$$MSE\left(\eta \right)=\frac{1}{10000}\sum_{i=1}^{10000}{\left(\hat{\eta }-\eta \right)}^{2},MSE\left(\lambda \right)=\frac{1}{10000}\sum_{i=1}^{10000}{\left(\hat{\lambda }-\lambda \right)}^{2}\;and\;MSE\left(\vartheta \right)=\frac{1}{10000}\sum_{i=1}^{10000}{\left(\hat{\vartheta }-\vartheta \right)}^{2}\text{.}$$

The results of the simulation study are shown in Table 2, Table 3, Table 4, Table 5, Table 6, Table 7, Table 8 listed below. A notable finding from these tables is that all estimators for the proposed distribution parameters have excellent reliability and are close to their real parameter values. As expected, the AB and MSEs decrease as the sample size grows across all parameter settings. Table 8 shows the estimator's cumulative rankings. This table clearly shows the weighted least squares approach is the most capable estimator among the five options, with the lowest score of 58.5.

**Table 2:** The ABs and MSEs for different estimates at η=0.5, λ=0.5 and ϑ=0.5

		MLE		ADE		CVME		OLSE		WLSE	
n	Para	AB	MSE	AB	MSE	AB	MSE	AB	MSE	AB	MSE
20	η	0.4395^{5}^	1.8989^{5}^	0.1652^{2}^	0.9132^{2}^	0.1982^{4}^	0.9293^{3}^	0.1436^{1}^	0.8324^{1}^	0.1708^{3}^	0.9500^{4}^
	λ	0.0823^{1}^	0.3058^{5}^	0.1453^{3}^	0.1732^{2}^	0.0995^{2}^	0.1800^{3}^	0.1643^{5}^	0.2085^{4}^	0.1531^{4}^	0.1677^{1}^
	ϑ	0.0295^{3}^	0.0291^{5}^	0.0234^{2}^	0.0141^{1}^	0.0070^{1}^	0.0201^{4}^	0.0358^{5}^	0.0170^{3}^	0.0335^{4}^	0.0154^{2}^
∑Ranks		24^{5}^		12^{1}^		17^{2}^		19^{4}^		18^{3}^	
50	η	0.3470^{5}^	1.1838^{5}^	0.1160^{3}^	0.5101^{3}^	0.0822^{2}^	0.3557^{2}^	0.0500^{1}^	0.2903^{1}^	0.1527^{4}^	0.6619^{4}^
	λ	0.0464^{1}^	0.2233^{5}^	0.0836^{4}^	0.0746^{2}^	0.0775^{3}^	0.0910^{4}^	0.1025^{5}^	0.0891^{3}^	0.0749^{2}^	0.0685^{1}^
	ϑ	0.0194^{4}^	0.0136^{5}^	0.0151^{2}^	0.0081^{2}^	0.0057^{1}^	0.0103^{4}^	0.0234^{5}^	0.0093^{3}^	0.0154^{3}^	0.0079^{1}^
∑Ranks		25^{5}^		16^{2.5}^		16^{2.5}^		18^{4}^		15^{1}^	
100	η	0.2583^{5}^	0.7729^{5}^	0.0512^{2}^	0.2610^{3}^	0.0636^{3}^	0.2336^{2}^	0.0386^{1}^	0.2024^{1}^	0.1077^{4}^	0.3985^{4}^
	λ	0.0147^{1}^	0.0941^{5}^	0.0720^{4}^	0.0543^{2}^	0.0565^{3}^	0.0608^{3}^	0.0738^{5}^	0.0607^{4}^	0.0522^{2}^	0.0488^{1}^
	ϑ	0.0144^{4}^	0.0080^{5}^	0.0134^{3}^	0.0063^{2}^	0.0043^{1}^	0.0073^{4}^	0.0149^{5}^	0.0069^{3}^	0.0079^{2}^	0.0061^{1}^
∑Ranks		25^{5}^		16^{2.5}^		16^{2.5}^		19^{4}^		14^{1}^	
200	η	0.1641^{5}^	0.4059^{5}^	0.0516^{3}^	0.1589^{3}^	0.0479^{2}^	0.1332^{2}^	0.0307^{1}^	0.1192^{1}^	0.0908^{4}^	0.2290^{4}^
	λ	0.0005^{1}^	0.0287^{1}^	0.0374^{4}^	0.0316^{3}^	0.0347^{3}^	0.0365^{5}^	0.0457^{5}^	0.0359^{4}^	0.0234^{2}^	0.0292^{2}^
	ϑ	0.0095^{5}^	0.0043^{3}^	0.0060^{3}^	0.0039^{1}^	0.0030^{2}^	0.0047^{5}^	0.0094^{4}^	0.0044^{4}^	0.0014^{1}^	0.0040^{2}^
∑Ranks		20^{5}^		17^{2}^		19^{3.5}^		19^{3.5}^		15^{1}^	
300	η	0.1242^{5}^	0.2354^{5}^	0.0383^{2}^	0.1058^{3}^	0.0422^{3}^	0.1000^{2}^	0.0344^{1}^	0.0948^{1}^	0.0689^{4}^	0.1376^{4}^
	λ	0.0072^{1}^	0.0198^{1}^	0.0265^{4}^	0.0227^{3}^	0.0230^{3}^	0.0274^{5}^	0.0294^{5}^	0.0271^{4}^	0.0136^{2}^	0.0212^{2}^
	ϑ	0.0090^{5}^	0.0030^{3}^	0.0045^{3}^	0.0029^{2}^	0.0014^{2}^	0.0036^{5}^	0.0053^{4}^	0.0034^{4}^	0.0001^{1}^	0.0028^{1}^
∑Ranks		20^{4.5}^		17^{2}^		20^{4.5}^		19^{3}^		14^{1}^

**Table 3:** The ABs and MSEs for different estimates at η=0.5, λ=0.5 and ϑ=1.0

		MLE		ADE		CVME		OLSE		WLSE	
n	Para	AB	MSE	AB	MSE	AB	MSE	AB	MSE	AB	MSE
20	η	0.4214^{5}^	1.8890^{5}^	0.1607^{2}^	0.9241^{3}^	0.1777^{4}^	0.8069^{2}^	0.1462^{1}^	0.7623^{1}^	0.1661^{3}^	0.9564^{4}^
	λ	0.0958^{1}^	0.3396^{5}^	0.1445^{3}^	0.1535^{3}^	0.0969^{2}^	0.1499^{2}^	0.1520^{5}^	0.1629^{4}^	0.1519^{4}^	0.1479^{1}^
	ϑ	0.0629^{3}^	0.1273^{5}^	0.0465^{2}^	0.0633^{1}^	0.0111^{1}^	0.0834^{4}^	0.0682^{5}^	0.0705^{3}^	0.0681^{4}^	0.0645^{2}^
∑Ranks		24^{5}^		14^{1}^		15^{2}^		19^{4}^		18^{3}^	
50	η	0.3363^{5}^	1.2268^{5}^	0.0820^{3}^	0.4782^{3}^	0.0819^{2}^	0.4493^{2}^	0.0507^{1}^	0.4041^{1}^	0.1234^{4}^	0.6380^{4}^
	λ	0.0511^{1}^	0.2331^{5}^	0.0884^{4}^	0.0759^{2}^	0.0729^{2}^	0.0837^{4}^	0.0993^{5}^	0.0834^{3}^	0.0784^{3}^	0.0688^{1}^
	ϑ	0.0419^{5}^	0.0641^{5}^	0.0290^{3}^	0.0318^{2}^	0.0049^{1}^	0.0405^{4}^	0.0408^{4}^	0.0366^{3}^	0.0284^{2}^	0.0315^{1}^
∑Ranks		26^{5}^		17^{3.5}^		15^{1.5}^		17^{3.5}^		15^{1.5}^	
100	η	0.2884^{5}^	0.7789^{5}^	0.0792^{2}^	0.2561^{3}^	0.0805^{3}^	0.2268^{2}^	0.0607^{1}^	0.1982^{1}^	0.1314^{4}^	0.3490^{4}^
	λ	0.0002^{1}^	0.0828^{5}^	0.0498^{4}^	0.0487^{2}^	0.0375^{3}^	0.0520^{4}^	0.0530^{5}^	0.0515^{3}^	0.0305^{2}^	0.0437^{1}^
	ϑ	0.0415^{5}^	0.0355^{5}^	0.0145^{3}^	0.0238^{2}^	0.0013^{1}^	0.0263^{4}^	0.0212^{4}^	0.0243^{3}^	0.0037^{2}^	0.0228^{1}^
∑Ranks		26^{5}^		16^{2}^		17^{3.5}^		17^{3.5}^		14^{1}^	
200	η	0.1775^{5}^	0.4246^{5}^	0.0562^{2}^	0.1700^{3}^	0.0615^{3}^	0.1420^{2}^	0.0464^{1}^	0.1275^{1}^	0.1021^{4}^	0.2666^{4}^
	λ	0.0017^{1}^	0.0416^{5}^	0.0422^{4}^	0.0329^{2}^	0.0350^{3}^	0.0384^{4}^	0.0448^{5}^	0.0378^{3}^	0.0260^{2}^	0.0298^{1}^
	ϑ	0.0187^{5}^	0.0181^{3}^	0.0166^{3}^	0.0165^{2}^	0.0065^{2}^	0.0201^{5}^	0.0183^{4}^	0.0189^{4}^	0.0061^{1}^	0.0162^{1}^
∑Ranks		24^{5}^		16^{2}^		19^{4}^		18^{3}^		13^{1}^	
300	η	0.1156^{5}^	0.2469^{5}^	0.0576^{3}^	0.1308^{3}^	0.0512^{2}^	0.1133^{2}^	0.0406^{1}^	0.1044^{1}^	0.0740^{4}^	0.1346^{4}^
	λ	0.0003^{1}^	0.0230^{1}^	0.0243^{3}^	0.0257^{3}^	0.0247^{4}^	0.0296^{5}^	0.0319^{5}^	0.0294^{4}^	0.0146^{2}^	0.0242^{2}^
	ϑ	0.0149^{5}^	0.0136^{3}^	0.0047^{3}^	0.0135^{2}^	0.0020^{2}^	0.0158^{5}^	0.0102^{4}^	0.0153^{4}^	0.0018^{1}^	0.0133^{1}^
∑Ranks		20^{4.5}^		17^{2}^		20^{4.5}^		19^{3}^		14^{1}^

**Table 4:** The ABs and MSEs for different estimates at η=0.5, λ=0.5 and ϑ=1.5

		MLE		ADE		CVME		OLSE		WLSE	
n	Para	AB	MSE	AB	MSE	AB	MSE	AB	MSE	AB	MSE
20	η	0.3907^{5}^	1.8327^{5}^	0.1931^{1}^	1.0600^{2}^	0.2210^{3}^	1.0356^{1}^	0.2224^{4}^	1.1112^{3}^	0.2106^{2}^	1.1362^{4}^
	λ	0.1032^{2}^	0.3593^{5}^	0.1222^{3}^	0.1337^{1}^	0.0817^{1}^	0.1495^{3}^	0.1294^{4}^	0.1577^{4}^	0.1322^{5}^	0.1404^{2}^
	ϑ	0.0784^{3}^	0.2757^{5}^	0.0518^{2}^	0.1291^{1}^	0.0386^{1}^	0.1917^{4}^	0.0775^{4}^	0.1551^{3}^	0.0792^{5}^	0.1467^{2}^
∑Ranks		25^{5}^		10^{1}^		13^{2}^		22^{4}^		20^{3}^	
50	η	0.3465^{5}^	1.3235^{5}^	0.1247^{2}^	0.6100^{3}^	0.1249^{3}^	0.5024^{2}^	0.1010^{1}^	0.4718^{1}^	0.1484^{4}^	0.6799^{4}^
	λ	0.0664^{1}^	0.2495^{5}^	0.0900^{4}^	0.0761^{2}^	0.0701^{2}^	0.0853^{4}^	0.0947^{5}^	0.0848^{3}^	0.0812^{3}^	0.0718^{1}^
	ϑ	0.0390^{2}^	0.1326^{5}^	0.0532^{4}^	0.0765^{2}^	0.0134^{1}^	0.0918^{4}^	0.0646^{5}^	0.0850^{3}^	0.0513^{3}^	0.0764^{1}^
∑Ranks		23^{4}^		17^{2}^		18^{3.5}^		18^{3.5}^		16^{1}^	
100	η	0.2751^{5}^	0.8413^{5}^	0.0876^{3}^	0.3567^{3}^	0.0853^{2}^	0.2982^{2}^	0.0693^{1}^	0.2796^{1}^	0.1156^{4}^	0.4025^{4}^
	λ	0.0152^{1}^	0.0931^{5}^	0.0588^{4}^	0.0486^{2}^	0.0454^{3}^	0.0532^{4}^	0.0593^{5}^	0.0523^{3}^	0.0448^{2}^	0.0450^{1}^
	ϑ	0.0404^{5}^	0.0762^{5}^	0.0318^{3}^	0.0519^{1}^	0.0070^{1}^	0.0598^{4}^	0.0351^{4}^	0.0557^{3}^	0.0196^{2}^	0.0520^{2}^
∑Ranks		26^{5}^		16^{2.5}^		16^{2.5}^		17^{4}^		15^{1}^	
200	η	0.2101^{5}^	0.4973^{5}^	0.0608^{2}^	0.1484^{3}^	0.0682^{3}^	0.1360^{2}^	0.0530^{1}^	0.1237^{1}^	0.0817^{4}^	0.1546^{4}^
	λ	0.0136^{1}^	0.0272^{1}^	0.0302^{4}^	0.0302^{3}^	0.0224^{3}^	0.0342^{4}^	0.0327^{5}^	0.0336^{5}^	0.0180^{2}^	0.0279^{2}^
	ϑ	0.0435^{5}^	0.0400^{4}^	0.0110^{3}^	0.0351^{2}^	0.0033^{2}^	0.0411^{5}^	0.0150^{4}^	0.0386^{3}^	0.0003^{1}^	0.0335^{1}^
∑Ranks		21^{5}^		17^{2}^		19^{3.5}^		19^{3.5}^		14^{1}^	
300	η	0.0981^{5}^	0.1841^{5}^	0.0466^{1}^	0.0977^{3}^	0.0587^{4}^	0.0990^{4}^	0.0479^{2}^	0.0911^{1}^	0.0567^{3}^	0.0954^{2}^
	λ	0.0033^{1}^	0.0191^{2}^	0.0182^{4}^	0.0208^{3}^	0.0120^{3}^	0.0260^{5}^	0.0192^{5}^	0.0254^{4}^	0.0103^{2}^	0.0188^{1}^
	ϑ	0.0243^{5}^	0.0258^{3}^	0.0035^{2}^	0.0252^{2}^	0.0099^{4}^	0.0331^{5}^	0.0028^{1}^	0.0313^{4}^	0.0037^{3}^	0.0235^{1}^
∑Ranks		21^{4}^		15^{2}^		25^{5}^		17^{3}^		12^{1}^

**Table 5:** The ABs and MSEs for different estimates at η=0.5, λ=1.5 and ϑ=0.5

		MLE		ADE		CVME		OLSE		WLSE	
n	Para	AB	MSE	AB	MSE	AB	MSE	AB	MSE	AB	MSE
20	η	0.4987^{5}^	2.0742^{5}^	0.3306^{2}^	1.5011^{2}^	0.3916^{4}^	1.6146^{3}^	0.3208^{1}^	1.4612^{1}^	0.3589^{3}^	1.6613^{4}^
	λ	0.0076^{1}^	0.5775^{5}^	0.0876^{5}^	0.2953^{4}^	0.0115^{2}^	0.2805^{3}^	0.0740^{4}^	0.2723^{2}^	0.0724^{3}^	0.2637^{1}^
	ϑ	0.0364^{5}^	0.0311^{5}^	0.0080^{1}^	0.0144^{1}^	0.0252^{4}^	0.0216^{4}^	0.0200^{3}^	0.0169^{3}^	0.0169^{2}^	0.0152^{2}^
∑Ranks		26^{5}^		15^{2.5}^		20^{4}^		14^{1}^		15^{2.5}^	
50	η	0.3524^{5}^	1.2461^{5}^	0.1323^{1}^	0.5619^{1}^	0.1803^{4}^	0.6474^{3}^	0.1428^{2}^	0.6170^{2}^	0.1541^{3}^	0.6651^{4}^
	λ	0.0082^{1}^	0.3730^{5}^	0.0710^{5}^	0.1899^{3}^	0.0256^{2}^	0.2011^{4}^	0.0585^{3}^	0.1835^{2}^	0.0588^{4}^	0.1705^{1}^
	ϑ	0.0233^{5}^	0.0146^{5}^	0.0070^{1}^	0.0084^{2}^	0.0091^{3}^	0.0104^{4}^	0.0109^{4}^	0.0089^{3}^	0.0088^{2}^	0.0081^{1}^
∑Ranks		26^{5}^		13^{1}^		20^{4}^		16^{3}^		15^{2}^	
100	η	0.2517^{5}^	0.6978^{5}^	0.0749^{3}^	0.2755^{3}^	0.0745^{2}^	0.2675^{2}^	0.0501^{1}^	0.2285^{1}^	0.1139^{4}^	0.4166^{4}^
	λ	0.0352^{2}^	0.1685^{5}^	0.0536^{4}^	0.1258^{2}^	0.0426^{3}^	0.1391^{4}^	0.0625^{5}^	0.1321^{3}^	0.0294^{1}^	0.1090^{1}^
	ϑ	0.0165^{5}^	0.0071^{5}^	0.0081^{3}^	0.0054^{2}^	0.0026^{1}^	0.0068^{4}^	0.0131^{4}^	0.0063^{3}^	0.0056^{2}^	0.0048^{1}^
∑Ranks		27^{5}^		17^{3.5}^		16^{2}^		17^{3.5}^		13^{1}^	
200	η	0.2053^{5}^	0.5394^{5}^	0.0751^{2}^	0.2069^{3}^	0.0816^{3}^	0.2039^{2}^	0.0623^{1}^	0.1793^{1}^	0.1064^{4}^	0.2642^{4}^
	λ	0.0353^{5}^	0.1069^{4}^	0.0280^{3}^	0.0956^{2}^	0.0161^{2}^	0.1085^{5}^	0.0308^{4}^	0.1043^{3}^	0.0002^{1}^	0.0846^{1}^
	ϑ	0.0117^{5}^	0.0046^{3}^	0.0043^{3}^	0.0039^{2}^	0.0003^{1}^	0.0049^{5}^	0.0066^{4}^	0.0047^{4}^	0.0008^{2}^	0.0036^{1}^
∑Ranks		27^{5}^		15^{2}^		18^{4}^		17^{3}^		13^{1}^	
300	η	0.1534^{5}^	0.3304^{5}^	0.0484^{1}^	0.1026^{1}^	0.0709^{4}^	0.1208^{4}^	0.0563^{2}^	0.1092^{3}^	0.0677^{3}^	0.1033^{2}^
	λ	0.0468^{5}^	0.0694^{3}^	0.0112^{4}^	0.0632^{2}^	0.0020^{1}^	0.0807^{5}^	0.0097^{3}^	0.0760^{4}^	0.0056^{2}^	0.0607^{1}^
	ϑ	0.0127^{5}^	0.0030^{3}^	0.0010^{1}^	0.0028^{2}^	0.0032^{4}^	0.0039^{5}^	0.0015^{2}^	0.0037^{4}^	0.0022^{3}^	0.0027^{1}^
∑Ranks		26^{5}^		11^{1}^		23^{4}^		18^{3}^		12^{2}^

**Table 6:** The ABs and MSEs for different estimates at η=1.5, λ=1.5 and ϑ=0.5

		MLE		ADE		CVME		OLSE		WLSE	
n	Para	AB	MSE	AB	MSE	AB	MSE	AB	MSE	AB	MSE
20	η	0.7510^{1}^	3.4042^{1}^	0.8897^{4}^	4.5194^{3}^	0.9656^{5}^	4.4944^{2}^	0.8705^{3}^	4.5441^{4}^	0.8363^{2}^	4.5533^{5}^
	λ	0.0467^{1}^	0.3154^{2}^	0.0963^{4}^	0.3378^{4}^	0.0472^{2}^	0.3059^{1}^	0.0901^{3}^	0.3208^{3}^	0.1175^{5}^	0.3538^{5}^
	ϑ	0.0253^{3}^	0.0136^{2}^	0.0135^{1}^	0.0130^{1}^	0.0151^{2}^	0.0184^{5}^	0.0316^{5}^	0.0156^{4}^	0.0313^{4}^	0.0143^{3}^
∑Ranks		10^{1}^		17^{2.5}^		17^{2.5}^		22^{4}^		24^{5}^	
50	η	0.6217^{1}^	2.9064^{1}^	0.7093^{3}^	3.5932^{3}^	0.8252^{5}^	3.8645^{5}^	0.7505^{4}^	3.8516^{4}^	0.6455^{2}^	3.4026^{2}^
	λ	0.0222^{2}^	0.1936^{1}^	0.0563^{4}^	0.2284^{5}^	0.0195^{1}^	0.2012^{2}^	0.0548^{3}^	0.2243^{4}^	0.0645^{5}^	0.2167^{3}^
	ϑ	0.0071^{2}^	0.0056^{1}^	0.0139^{3}^	0.0064^{2}^	0.0053^{1}^	0.0072^{4}^	0.0255^{5}^	0.0075^{5}^	0.0197^{4}^	0.0066^{3}^
∑Ranks		8^{1}^		20^{3.5}^		20^{3.5}^		25^{5}^		19^{2}^	
100	η	0.4750^{1}^	2.1511^{1}^	0.5641^{3}^	2.6810^{3}^	0.6518^{5}^	2.9741^{5}^	0.6067^{4}^	2.9734^{4}^	0.4952^{2}^	2.4762^{2}^
	λ	0.0276^{3}^	0.1431^{1}^	0.0393^{3}^	0.1581^{4}^	0.0239^{1}^	0.1517^{2}^	0.0456^{4}^	0.1657^{5}^	0.0537^{5}^	0.1566^{3}^
	ϑ	0.0004^{1}^	0.0033^{1}^	0.0122^{3}^	0.0040^{2}^	0.0109^{2}^	0.0041^{3}^	0.0213^{5}^	0.0045^{5}^	0.0155^{4}^	0.0042^{4}^
∑Ranks		8^{1}^		18^{2.5}^		18^{2.5}^		27^{5}^		20^{4}^	
200	η	0.3432^{3}^	1.4553^{1}^	0.3413^{2}^	1.6473^{3}^	0.4068^{5}^	1.9799^{4}^	0.3873^{4}^	2.0419^{5}^	0.3252^{1}^	1.6268^{2}^
	λ	0.0057^{1}^	0.0826^{1}^	0.0279^{2}^	0.0999^{2}^	0.0311^{3}^	0.1095^{4}^	0.0473^{5}^	0.1216^{5}^	0.0372^{4}^	0.1058^{3}^
	ϑ	0.0002^{1}^	0.0017^{1}^	0.0091^{2}^	0.0022^{2}^	0.0117^{4}^	0.0026^{4}^	0.0180^{5}^	0.0030^{5}^	0.0113^{3}^	0.0024^{3}^
∑Ranks		8^{1}^		13^{2}^		24^{4}^		29^{5}^		16^{3}^	
300	η	0.2798^{3}^	1.0947^{5}^	0.2579^{2}^	1.0676^{4}^	0.3754^{4}^	1.5940^{1}^	0.3617^{5}^	1.6010^{2}^	0.2556^{1}^	1.0653^{3}^
	λ	0.0036^{1}^	0.0621^{1}^	0.0147^{2}^	0.0652^{3}^	0.0214^{4}^	0.0944^{4}^	0.0287^{5}^	0.0987^{5}^	0.0177^{3}^	0.0642^{2}^
	ϑ	0.0001^{1}^	0.0013^{1}^	0.0051^{2}^	0.0015^{3}^	0.0092^{4}^	0.0021^{4}^	0.0128^{5}^	0.0022^{5}^	0.0061^{3}^	0.0014^{2}^
∑Ranks		12^{1}^		16^{3}^		21^{4}^		27^{5}^		14^{2}^

**Table 7:** The ABs and MSEs for different estimates at η=1.5, λ=1.5 and ϑ=1.5

		MLE		ADE		CVME		OLSE		WLSE	
n	Para	AB	MSE	AB	MSE	AB	MSE	AB	MSE	AB	MSE
20	η	0.7229^{1}^	3.3753^{1}^	0.8879^{4}^	4.5787^{4}^	0.9525^{5}^	4.4860^{2}^	0.8822^{3}^	4.5522^{3}^	0.8476^{2}^	4.6206^{5}^
	λ	0.0466^{1}^	0.3091^{3}^	0.0909^{4}^	0.3312^{4}^	0.0467^{2}^	0.3020^{1}^	0.0765^{3}^	0.3029^{2}^	0.1089^{5}^	0.3397^{5}^
	ϑ	0.0750^{3}^	0.1248^{2}^	0.0426^{2}^	0.1145^{1}^	0.0279^{1}^	0.1378^{5}^	0.1016^{5}^	0.1271^{4}^	0.0971^{4}^	0.1252^{3}^
∑Ranks		11^{1}^		19^{3}^		16^{2}^		20^{4}^		24^{5}^	
50	η	0.6348^{1}^	2.9994^{1}^	0.7300^{4}^	3.7041^{4}^	0.7592^{5}^	3.6814^{3}^	0.7102^{3}^	3.7411^{5}^	0.6638^{2}^	3.4889^{2}^
	λ	0.0456^{1}^	0.2031^{1}^	0.0726^{3}^	0.2208^{4}^	0.0491^{2}^	0.2097^{2}^	0.0782^{4}^	0.2304^{5}^	0.0812^{5}^	0.2206^{3}^
	ϑ	0.0092^{1}^	0.0515^{1}^	0.0508^{3}^	0.0552^{2}^	0.0266^{2}^	0.0575^{4}^	0.0850^{5}^	0.0625^{5}^	0.0676^{4}^	0.0569^{3}^
∑Ranks		6^{1}^		20^{4}^		18^{2}^		27^{5}^		19^{3}^	
100	η	0.5544^{1}^	2.5865^{1}^	0.6357^{3}^	3.0442^{3}^	0.6872^{5}^	3.1751^{4}^	0.6640^{4}^	3.2379^{5}^	0.5796^{2}^	2.8107^{2}^
	λ	0.0369^{2}^	0.1517^{2}^	0.0442^{3}^	0.1586^{4}^	0.0308^{1}^	0.1531^{3}^	0.0475^{5}^	0.1657^{5}^	0.0456^{4}^	0.1478^{1}^
	ϑ	0.0055^{1}^	0.0315^{1}^	0.0384^{3}^	0.0345^{3}^	0.0332^{2}^	0.0368^{4}^	0.0630^{5}^	0.0403^{5}^	0.0434^{4}^	0.0332^{2}^
∑Ranks		8^{1}^		19^{3.5}^		19^{3.5}^		29^{5}^		15^{2}^	
200	η	0.3485^{2}^	1.6361^{1}^	0.3708^{3}^	1.8325^{3}^	0.4766^{5}^	2.2486^{4}^	0.4581^{4}^	2.2886^{5}^	0.3395^{1}^	1.6718^{2}^
	λ	0.0443^{2}^	0.1167^{2}^	0.0525^{5}^	0.1215^{4}^	0.0360^{1}^	0.1182^{3}^	0.0494^{4}^	0.1282^{5}^	0.0464^{3}^	0.1031^{1}^
	ϑ	0.0141^{1}^	0.0220^{2}^	0.0359^{4}^	0.0244^{3}^	0.0345^{3}^	0.0249^{4}^	0.0519^{5}^	0.0275^{5}^	0.0339^{2}^	0.0215^{1}^
∑Ranks		10^{1.5}^		22^{4}^		20^{3}^		28^{5}^		10^{1.5}^	
300	η	0.2701^{3}^	1.1620^{2}^	0.2580^{2}^	1.1767^{3}^	0.2975^{5}^	1.3707^{4}^	0.2848^{4}^	1.3991^{5}^	0.2537^{1}^	1.1291^{1}^
	λ	0.0208^{1}^	0.0752^{3}^	0.0286^{4}^	0.0744^{2}^	0.0293^{3}^	0.0836^{4}^	0.0376^{5}^	0.0880^{5}^	0.0231^{2}^	0.0671^{1}^
	ϑ	0.0096^{1}^	0.0133^{2}^	0.0257^{3}^	0.0143^{3}^	0.0322^{4}^	0.0171^{4}^	0.0433^{5}^	0.0185^{5}^	0.0245^{2}^	0.0131^{1}^
∑Ranks		12^{2}^		17^{3}^		24^{4}^		29^{5}^		8^{1}^

**Table 8:** Partial and overall rankings of the five estimation methods for various parameter combinations.

Parameter	n	MLE	ADE	CVME	OLSE	WLSE
η=0.5 λ=0.5 ϑ=0.5	20	5	1	2	4	3
	50	5	2.5	2.5	4	1
	100	5	2.5	2.5	4	1
	200	5	2	3.5	3.5	1
	300	4.5	2	4.5	3	1
η=0.5 λ=0.5 ϑ=1.0	20	5	1	2	4	3
	50	5	3.5	1.5	3.5	1.5
	100	5	2	3.5	3.5	1
	200	5	2	4	3	1
	300	4.5	2	4.5	3.	1
η=0.5 λ=0.5 ϑ=1.5	20	5	1	2	4	3
	50	4	2	3.5	3.5	1
	100	5	2.5	2.5	4	1
	200	5	2	3.5	3.5	1
	300	4	2	5	3	1
η=0.5 λ=1.5 ϑ=0.5	20	5	2.5	4	1	2.5
	50	5	1	4	3	2
	100	5	3.5	2	3.5	1
	200	5	2	4	3	1
	300	5	1	4	3	2
η=1.5 λ=1.5 ϑ=0.5	20	1	2.5	2.5	4	5
	50	1	3.5	3.5	5	2
	100	1	2.5	2.5	5	4
	200	1	2	4	5	3
	300	1	3	4	5	2
η=1.5 λ=1.5 ϑ=1.5	20	1	3	2	4	5
	50	1	4	2	5	3
	100	1	3.5	3.5	5	2
	200	1.5	4	3	5	1.5
	300	2	3	4	5	1
∑Ranks		108.5	71	96	115	58.5
Overall Rank		4	2	3	5	1

## 5 Application

Real-life datasets are used to show the PQXg distribution's applicability. The maximum likelihood approach is used to estimate the parameters of the proposed model based on the datasets. The PQXg distribution's performance is also compared to various other distributions, including the quasi-Xgamma (QXg), Xgamma (Xg), Lindley (L), Exponentiated Lindley (EL), and Weighted Lindley (WL). The Akaike Information Criterion (AIC), log-likelihood (Log-Lik) Bayesian Information Criterion (BIC), and Kolmogorov Smirnov (KS) are used to compare the results. The model with the lowest AIC, BIC, and KS test statistic values and the highest Log-Lik and KS p-value among competing models is regarded as the best fit.

The first dataset is about the failure time of 50 components [29]. The observations are given as: 15.080, 14.730, 13.880, 11.020, 10.940, 9.337, 8.022, 7.904, 7.896, 6.816, 6.274, 4.893, 4.534, 4.393, 4.073, 3.931, 3.704, 3.625, 3.147, 3.076, 3.058, 2.804, 2.054, 2.006, 1.600, 1.228, 0.961, 0.645, 0.618, 0.590, 0.574, 0.570, 0.538, 0.381, 0.379, 0.262, 0.254, 0.192, 0.183, 0.148, 0.116, 0.114, 0.103, 0.102, 0.086, 0.078, 0.074, 0.061, 0.058, and 0.036.

The second dataset is about the breaking strength of carbon fibers of 50 mm length [30]. The observations are; 4.90, 4.70, 4.42, 4.38, 4.20, 3.75, 3.70, 3.68, 3.65, 3.60, 3.56, 3.39, 3.39, 3.33, 3.31, 3.31, 3.28, 3.27, 3.22, 3.22, 3.19, 3.15, 3.15, 3.11, 3.11, 3.09, 2.97, 2.96, 2.95, 2.93, 2.88, 2.87, 2.85, 2.82, 2.81, 2.79, 2.74, 2.73, 2.67, 2.59, 2.56, 2.55, 2.55, 2.53, 2.50, 2.48, 2.43, 2.41, 2.35, 2.12, 2.05, 2.03, 2.03, 1.89, 1.87, 1.84, 1.80, 1.69, 1.61, 1.61, 1.57, 1.47, 1.25, 1.08, 0.85, and 0.39.

**Fig. 3:**
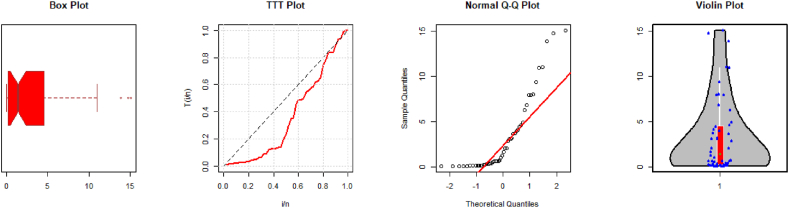
Descriptive plots for the first dataset.

**Fig. 4:**
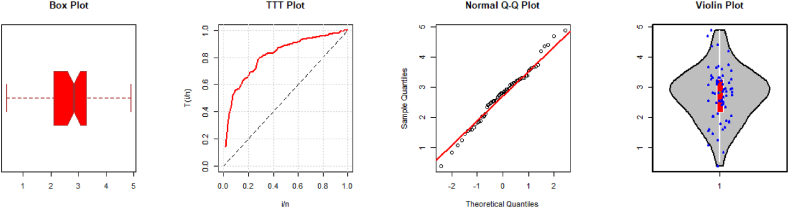
Descriptive plots for the second dataset.

Box and TTT (total test time) plots for both datasets are presented in Fig. 3, Fig. 4. Some descriptive statistics such as minimum observation (Ymin), first quartile ($Q_{1}$), mean, median, third quartile ($Q_{3}$), standard deviation (SD), maximum value (Ymax), skewness, and kurtosis are given in Table 9.

**Table 9:** Some descriptive measures of both datasets.

	$Y_{\min}$	$Q_{1}$	Median	Mean	$Q_{3}$	$Y_{\max}$	SD	Skewness	Kurtosis
**Data 1**	0.036	0.2075	1.414	3.343	4.4988	15.08	4.182	1.417	4.085
**Data 2**	0.390	2.178	2.835	2.760	3.277	4.90	0.891	−0.1315	3.2230

**Fig. 5:**
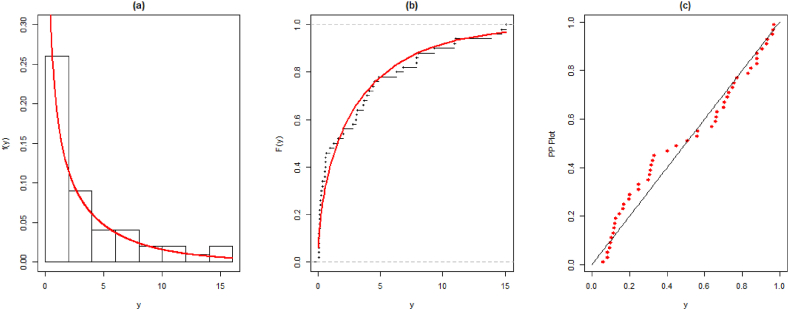
Fitted and empirical PDF, CDF, and PP plots for the first dataset.

**Fig. 6:**
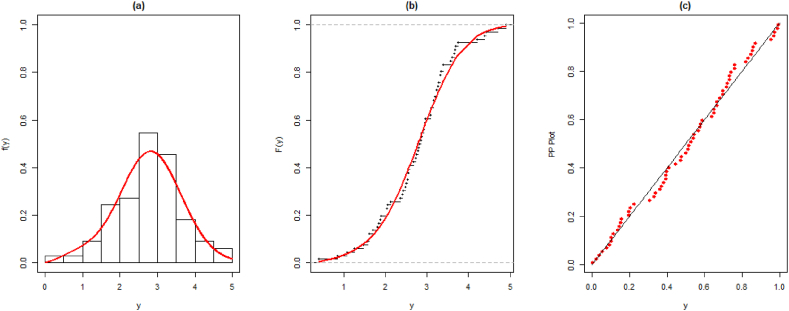
Fitted and empirical PDF, CDF, and PP plots for the second dataset.

The MLEs of all considered models are given in Table 10, Table 11. The fitted PDF and CDF for both datasets are given in Fig. 5, Fig. 6.

**Table 10:** The parameter estimates, and standard error (SE) for the first data set along with goodness-of-fit measures.

Model	Parameters		-Log-Lik	AIC	BIC	KS	
	Estimates	SEs				Statistic	p-value
**PQXg**	λ = 0.89741	0.07644	101.375	208.751	214.487	0.13053	0.33250
	η = 1.89614	2.27175					
	ϑ = 0.67359	0.07914					
**QXg**	λ = 0.43192	0.0765	108.545	221.091	224.915	0.27020	0.00102
	η = 3.50260	2.2840					
**EL**	λ = 0.29648	0.05411	103.981	211.962	215.786	0.16303	0.12510
	η = 0.41128	0.07111					
**WL**	λ = 0.28588	0.04755	105.138	214.277	218.101	0.18008	0.06856
	η = 0.38317	0.07689					
**Xg**	λ = 0.61729	0.05858	115.990	233.980	235.892	0.32980	0.00002
**Lindley**	λ = 0.49873	0.05131	120.178	242.355	244.267	0.34053	0.00001

**Table 11:** The parameter estimates, and standard error (SE) for the second data set along with goodness-of-fit measures.

Model	Parameters		-Log-Lik	AIC	BIC	KS	
	Estimates	SEs				Statistic	p-value
**PQXg**	λ = 0.22259	0.12466	85.0721	176.144	182.713	0.07074	0.89590
	η = 0.18145	0.18728					
	ϑ = 2.31438	0.40385					
**QXg**	λ = 1.15156	0.08704	100.159	204.319	208.698	0.21515	0.00444
	η = 0.08171	0.02555					
**EL**	λ = 1.24607	0.10900	93.7969	191.593	195.973	0.14697	0.11550
	η = 7.04169	1.67331					
**WL**	λ = 2.79536	0.47783	90.9291	185.858	190.237	0.13191	0.20090
	η = 6.99868	1.27125					
**Xg**	λ = 0.82103	0.06612	123.719	249.438	251.628	0.32796	0.00000
**Lindley**	λ = 0.59026	0.05324	122.384	246.768	248.957	0.29771	0.00002

The above Tables and graphs show that the introduced model provides better-fitted values as compared to QXg, Xg, L, EL, and WL.

## 6 Conclusion

This study introduces a new three-parameter flexible distribution which represents a substantial improvement in the field of statistics. The probability model exhibits properties that provide academics and practitioners with a more adaptive and versatile tool for modeling real-life datasets in a variety of fields including engineering, finance, agriculture, and natural sciences. Some mathematical properties include incomplete and complete moments, moment generating function, quantile function, Rényi, and Tsallis entropies, survival and hazard functions, mean residual life, stress-strength reliability, mean inactivity time, and strong mean inactivity time. The parameters of the derived were estimated using five different estimation methods. The performance of the derived estimators and best estimation method is identified via a comprehensive simulation study. The WLS estimation method efficiently estimates the PQXg distribution parameters. In the end, the proposed distribution is applied to two real-life data sets which appear to be better-fitted distributions as compared to the other competitive models.

## 7 Future work

There may be various possible areas of investigation connected to the new three-parameter distribution in the future. Here are several examples:

Further analysis of probability model features: Scientists may investigate the mathematical aspects of the suggested distribution in further depth. This might include developing new reliability characteristics, and characterizations, or investigating the relationship with other probability models.

• Parameter Estimation: Future work might concentrate on the Bayesian estimation technique for the parameters of the proposed distribution. Researchers may additionally investigate the estimators' qualities, such as asymptotic behavior and efficiency.
• Neutrosophic Extension: A neutrosophic extension can also be introduced to analyzed datasets with indeterminacy.
• Regression modeling: The new distribution might be used in regression models to assess its efficacy in predicting or explaining variable connections.

## CRediT authorship contribution statement

**Abdullah M. Alomair:** Supervision, Funding acquisition, Methodology. **Ayesha Babar:** Writing – original draft. **Muhammad Ahsan-ul-Haq:** Writing – review & editing, Writing – original draft. **Saadia Tariq:** Supervision.

## Data availability

“All the data sets used in this paper are available within the manuscript”.

## Use of AI tools declaration

“The authors declare they have not used Artificial Intelligence (AI) tools in the creation of this article”.

## Declaration of competing interest

The authors declare that they have no known competing financial interests or personal relationships that could have appeared to influence the work reported in this paper.
